# The neural bases of host plant selection in a Neuroecology framework

**DOI:** 10.3389/fphys.2015.00229

**Published:** 2015-08-12

**Authors:** Carolina E. Reisenman, Jeffrey A. Riffell

**Affiliations:** ^1^Department of Molecular and Cell Biology, University of CaliforniaBerkeley, CA, USA; ^2^Department of Biology, University of WashingtonSeattle, WA, USA

**Keywords:** Neuroecology, insect olfaction, oviposition, moths, neurons

## Abstract

Understanding how animals make use of environmental information to guide behavior is a fundamental problem in the field of neuroscience. Similarly, the field of ecology seeks to understand the role of behavior in shaping interactions between organisms at various levels of organization, including population-, community- and even ecosystem-level scales. Together, the newly emerged field of “Neuroecology” seeks to unravel this fundamental question by studying both the function of neurons at many levels of the sensory pathway and the interactions between organisms and their natural environment. The interactions between herbivorous insects and their host plants are ideal examples of Neuroecology given the strong ecological and evolutionary forces and the underlying physiological and behavioral mechanisms that shaped these interactions. In this review we focus on an exemplary herbivorous insect within the Lepidoptera, the giant sphinx moth *Manduca sexta*, as much is known about the natural behaviors related to host plant selection and the involved neurons at several level of the sensory pathway. We also discuss how herbivore-induced plant odorants and secondary metabolites in floral nectar in turn can affect moth behavior, and the underlying neural mechanisms.

## Herbivory and host specialization

Herbivory is a major evolutionary achievement in eukaryotic animals and in particular in insects, with nearly half of all existing species feeding on living plants (Gilbert, 1979). Lepidoptera (moths and butterflies) are the largest lineage of plant-feeding organisms, and their evolution is intimately related to the radiation of angiosperms in the Cretaceous (Grimaldi and Engel, 2005). The other large groups of phytophagous insects are found among the Coleoptera and include weevils, leaf beetles, and the long-horned beetles (Grimaldi and Engel, 2005).

The evolutionary processes that cause the diversification of herbivorous insects are not completely understood but host–plant interactions, and in particular plant chemistry, are thought to be a critical factor (Ehrlich and Raven, 1964; Jaenike, 1990; Whiteman and Jander, 2010). Plant volatiles contribute to sympatric speciation and reproductive isolation involving host plant shifts, such as those observed in races of the larch bud moth *Zyraphera diniana* having different host preferences (Emelianov et al., 2003; Syed et al., 2003), and in the apple maggot *Rhagoletis pomonella* (Linn et al., 2003; Olsson et al., 2006). Changes in host plant preferences can occur very fast, particularly in cases in which few genes participate in mediating host plant selections (Linn et al., 2003; Schoonhoven et al., 2005). However, for divergent plants that have converged on a similar chemical profile, the similarity can facilitate exploitation by related herbivores—an example of this occurs in checkerspot butterfly larvae *Euphydryas chalcedona*, which are stimulated by host plants that have iridoid glycosylates (Bowers, 1983).

While many herbivorous insects feed in many plant species, most herbivorous are specialists, with larvae feeding and adults ovipositing on a small number of closely related plant species, usually within the same family (Jaenike, 1990). Transitions from a generalist lifestyle to specialization are common, and it has been suggested that they resulted from genetically based trade-offs in offspring performance (Jaenike, 1990). Many morphological and physiological adaptations accompany host plant specialization, including detoxification mechanisms against plant defenses and sensory specializations for the detection of host-derived chemical (olfactory and taste) cues (Schoonhoven et al., 2005). In particular, the importance of the chemosensory system in host plant choice, along with the fact that specialists outnumber generalists, suggests that the evolution of insect–plant interactions is based on changes within the insect nervous system (Olsson et al., 2006), with such changes occurring before host plant shifts (e.g., Dekker et al., 2006; Lavista-Llanos et al., 2014).

In this review we focus on the neural mechanisms underlying host plant selection by moths in a “Neuroecological” context, that is, the function of neurons in an adaptive biologically relevant framework. Almost all of what we review here draws from studies in the exemplary giant sphinx moth *Manduca sexta* (Spinghidae, Lepidoptera), as we know much about both the anatomical and functional organization of its chemosensory system and about the olfactory cues that guide host finding in this crepuscular/nocturnal insect.

## Host plants chemical signals

Olfactory signals play decisive roles in the lives of adult moths, including *M. sexta*. Both sexes of this nocturnally active insect must find flowers on which to feed, males must find females, and gravid females must find the proper host plants on which to lay their eggs. A particularly well characterized olfactory-guided behavior of adult *M. sexta* (as well as of many other moth species) is the oriented flight response of males to the sex-pheromone blend released by conspecific females (Baker, 1990; Willis and Arbas, 1991). Corresponding neurophysiological studies have shown that specialized male-specific neurons at several levels of the olfactory pathway faithfully encode the pheromone signal and control male behavior (Schneiderman et al., 1986; Christensen et al., 1996; Heinbockel et al., 1999, 2004; Lei et al., 2002; Dacks et al., 2007; Riffell et al., 2008a).

**Figure 1:**
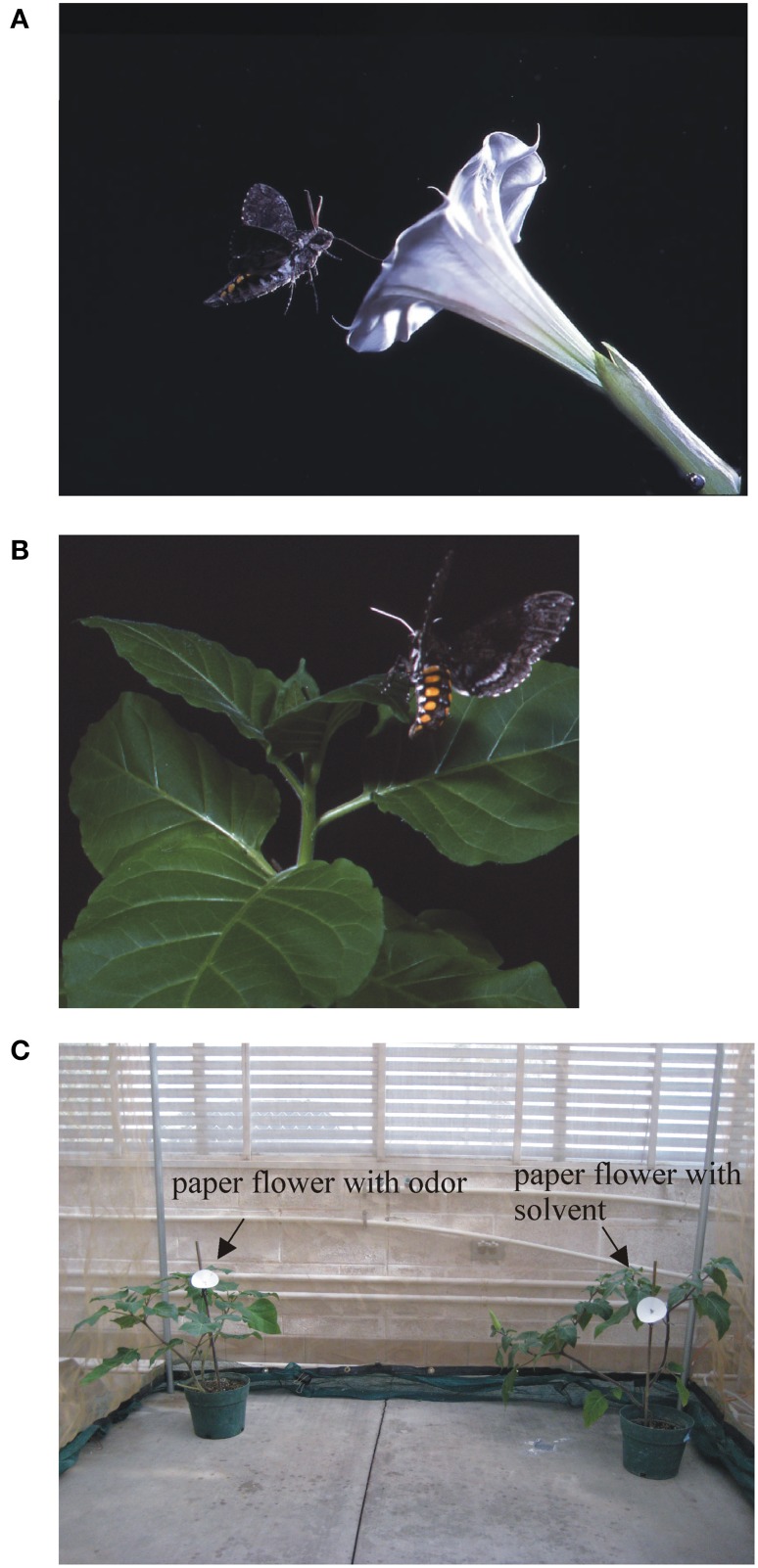
**(A) Adult ***M. sexta*** feeding on nectar from a flower from one of its favorite host plants, the jimsonweed ***Datura wrightii*****. **(B)** Females also use this plant as an oviposition resource (Picture by C. Hedgcock). **(C)** Experimental set-up used to study the oviposition behavior of females in the laboratory. Singly mated female are released in a flight cage in a dual-choice experiment. In this case, the insect is offered two plants, one with an artificial paper flower loaded with a synthetic odor (experimental) and the other loaded with the solvent (control).

How the olfactory system process information about feeding and oviposition resources, in contrast, only recently begun to be understood. Both sexes feed on nectar from flowers, but gravid females require host plants also as oviposition sites (Madden and Chamberlin, 1945; Reisenman et al., 2010; Figures 1A,B). Thus, females need specialized receptors and neurons for detecting appropriate host plants for oviposition. Like many other hawkmoths, *M. sexta* adults pollinate large, tubular, night-blooming white or pale flowers (Sparks, 1969, 1973; Alarcón et al., 2008) which produce large quantities of volatile organic compounds (VOCs) (Raguso and Willis, 2002; Raguso et al., 2003). In the Southwestern USA, the sacred daturas or jimsonweeds, *D. wrightii* and *D. discolor*, attract *M. sexta* as pollinators and also function as host plants for their larvae (Mira and Bernays, 2002; Figures 1A,B). Their flowers provide rich nectar sources to foraging adults (Raguso and Willis, 2002) and bloom once over a single night, but a plant can produce >200 flowers in one season. Floral odors and visual signals, alone or in combination, are innately attractive to adults of both sexes (Raguso and Willis, 2002; Goyret, 2010; Kaczorowski et al., 2012; Riffell and Alarcón, 2013), but only the simultaneous presence of both signals elicits nectar feeding (Ramaswamy, 1988; Raguso et al., 2003).

The selection of appropriate host plants for oviposition by gravid moths is undoubtedly crucial for the success of her larval offspring. In contrast to feeding, female *M. sexta* rely primarily on olfactory cues to locate and identify host plants for oviposition (Sparks, 1973; Ramaswamy, 1988; Reisenman et al., 2010; Späthe et al., 2013). As other Lepidoptera (Renwick and Chew, 1994), female *M. sexta* may contact host plants with their tarsi prior to oviposition (Yamamoto et al., 1969; Sparks, 1973), but this behavior is not a requirement (Mechaber et al., 2002). In general, the contribution of taste to host plant selection is not completely understood, although highly specialized taste receptors are present in specialist herbivores. Once the host plant is located, taste receptors mediate a sequence of behavioral events leading to egg lying (Schoonhoven et al., 2005).

As many herbivores, *M. sexta* moths are highly specialized. Larvae feed almost exclusively (but see Mira and Bernays, 2002; Mechaber et al., 2002) on plants of the family Solanaceae [e.g., native jimsonweeds (*Datura* sp.), native and cultivated species of tobacco (*Nicotiana* sp.), tomato, eggplant, pepper, etc., (Madden and Chamberlin, 1945; Yamamoto and Fraenkel, 1960; Tichenor and Seigler, 1980; el Campo et al., 2001)]. Although both nectar and leaves of some of these plants contain alkaloids which are toxic for many other insect species, moths have detoxification mechanisms that allow them to deal with these secondary compounds (Glendinning, 2002; Adler et al., 2006; Hare and Walling, 2006). Though plant defensive compounds can be toxic to specialist herbivores at high (unnatural) concentrations, on average, specialist herbivores are less negatively impacted than generalists (Berenbaum et al., 1989; Cornell and Hawkins, 2003). Thus, both chemosensory specialization and tolerance to toxic components are both crucial components for insect host plant specialization and coevolution.

### Herbivory and host plants: a dynamically changing olfactory environment

While specific olfactory cues from host plants are necessary for acceptance and egg laying by gravid females (Yamamoto and Fraenkel, 1960), the VOCs released by plants are not static, but are rather affected by the time of the day and other physiological and environmental factors (De Moraes et al., 2001). For instance, plants respond to herbivory with changes in plant chemistry and physiology that make them more resistant to further attack, such as the induction of toxic metabolites that can poison attacking herbivores or slow their growth (Karban and Baldwin, 1997; Baldwin and Preston, 1999). Plants also use indirect defenses, i.e., synthesize and release complex blends of VOCs that attract the natural enemies of the herbivores (De Moraes et al., 1998; Turlings et al., 1998; Baldwin and Preston, 1999; Paré and Tumlinson, 1999; Dicke and van Loop, 2000; Halitschke et al., 2000; Schnee et al., 2006). These VOCs, which include monoterpenes, sesquiterpenes, and aromatics (Paré and Tumlinson, 1999) are produced *de novo* (Paré and Tumlinson, 1997), systemically (De Moraes et al., 1998) and slowly (>24-h post-attack; Kessler and Baldwin, 2001), and are qualitatively different from the VOCs released by mechanically damaged plants (Paré and Tumlinson, 1999; Halitschke et al., 2000; De Moraes et al., 2001; Kessler and Baldwin, 2001; Reisenman et al., 2013). For instance, feeding by *M. sexta* larvae on *Nicotiana* sp. (tobacco) induces both direct and indirect defenses (Halitschke et al., 2000; De Moraes et al., 2001; Adler et al., 2006; McCall and Karban, 2006). In principle, a gravid female should avoid ovipositing in such induced plants, as they are likely to host insects that will compete with her offspring and also natural enemies attracted by the induced VOCs. Indeed, both *M. sexta* and *M. quinquemaculata* moths avoid ovipositing on larva-damaged plants (Kessler and Baldwin, 2001) in a plant-species specific manner (Reisenman et al., 2013; Späthe et al., 2013). Alternatively, as observed in other insect species, egg-lying by multiple females in nearby sites could reduce predation risk for each female's offspring (Jaenike, 1978). Also, in certain moth species, oviposition is deterred by VOCs emitted by larval frass (Anderson et al., 1992; Xu et al., 2006) and in *M. sexta* is affected by the presence of con-specific larvae (Reisenman et al., 2013).

Insect herbivores are agents of selection on plant defense traits because plant populations excluded from herbivory evolve lower resistance and higher competitive ability, but these populations can quickly regain increased resistance when re-exposed to the herbivore (Agrawal et al., 2012; Uesugi and Kessler, 2013; Sakata et al., 2014). Thus, moths will be experiencing a spatiotemporally changing landscape of suitable host plants, many of whom will vary in their induced chemical defenses, resources, and growth potential. For instance, induction of plant defense pathways in tomato or *Arabidopsis sp*. results in significant reduction in growth, physiological performance, and fitness (Cipollini, 2010; Corrado et al., 2011), all of which can indirectly affect the growth of the developing larvae. Moreover, this spatiotemporal complexity in the plant community—*via* induction of plant defenses—provide different kinds of information to the herbivore and the plant community: (1) for plants, it can reduce the probability of secondary attacks and provide host cues for the natural enemies of the herbivores, while providing information to and from neighboring plants; and (2) for the herbivore, it provides information about the suitability of the host plant with regards to its chemical defenses and its metabolic and physiological health.

The detection and decision-making ability of adult moths in response to the dynamic host plant chemical information is additionally affected when the host plants serve also as floral nectar resources. For instance, leaf herbivory can result in smaller flowers and fewer open flowers (Mothershead and Marquis, 2000; Adler et al., 2001), leading to lower amounts of floral VOC emissions and less pollinator visitation. Additionally, the biosynthetic pathways of inducible plant defenses can be constitutively expressed throughout the plant tissue. Thus, when damaged, there lies the potential that floral scent is modified (Heil and Ton, 2010). However, results from this hypothesis are mixed: in one study larvae damaged by *M. sexta* of sweet tobacco (*Nicotiana suaveolens*) did not significantly increase floral volatile production (Effmert et al., 2008), while in wild tomato plants (*S. peruvianum*) damaged by the same herbivore the floral blend significantly differed from that of non-damaged plants (Kessler and Halitschke, 2009). For other plant families, induction of plant defenses appears to be systemic and flowers can either produce VOCs *de novo* in response to herbivory (Loughrin et al., 1994; Röse and Tumlinson, 2004) or decrease emissions altogether (Pareja et al., 2012). Thus, the interplay between pollinator attraction and host plant defense provides a unique system to identify the cues and associated sensory mechanisms by which plants manipulate their interaction with insects.

### A naturalistic insect–plant interaction

In contrast with older studies which use artificially selected crops grown in simple agro-ecosystems (Harvey et al., 2015), much work in the last decade focused on more naturalistic insect–plant interactions. For instance, a particularly interesting mutually beneficial association exists in the Sonoran Desert in Southwest USA between *M. sexta* and the jimsonweed *D. wrightii*: while flowers are pollinated by adults (Alarcón et al., 2008; Riffell et al., 2008b), the plants serve as food resources for the larvae (Mechaber and Hildebrand, 2000; Figures 1A,B). Detoxification mechanisms (Glendinning, 2002) enable larvae to cope with herbivory-induced toxic secondary compounds (Adler et al., 2006; Hare and Walling, 2006), and plants cope with the negative effects of herbivory by quickly recovering after larval damage (Reisenman et al., 2013). Importantly, plants benefit from this association because jimsonweeds set more fruit by cross-pollination (Nunez-Farfan et al., 1996; Raguso et al., 2003) and plants are not completely defoliated, as eggs and larvae suffer high levels of parasitism in the field (Strauss and Agrawal, 1999; Kester et al., 2002; Mira and Bernays, 2002). Furthermore, feeding of larva in vegetative tissues, while causing changes in the vegetative VOC profile, does not affect the quantitative and qualitative composition of the floral VOCs that are necessary to mediate feeding attraction (Riffell et al., 2009b; Reisenman et al., 2013). Thus, this insect–plant interaction has been described as a non-obligatory mutualistic pollinator-herbivore association (Bronstein et al., 2009). In the next section we describe the neural pathway/s and substrates used by the moths to detect and locate suitable host plants. We argue that this exemplar insect–plant interaction illustrates an undeniable perspective: that neurobiological experimentation in a “Neuroecology” context has the most chances of helping discovering how neural circuits function to ultimately produce behavior.

## The moth olfactory pathway

**Figure 2:**
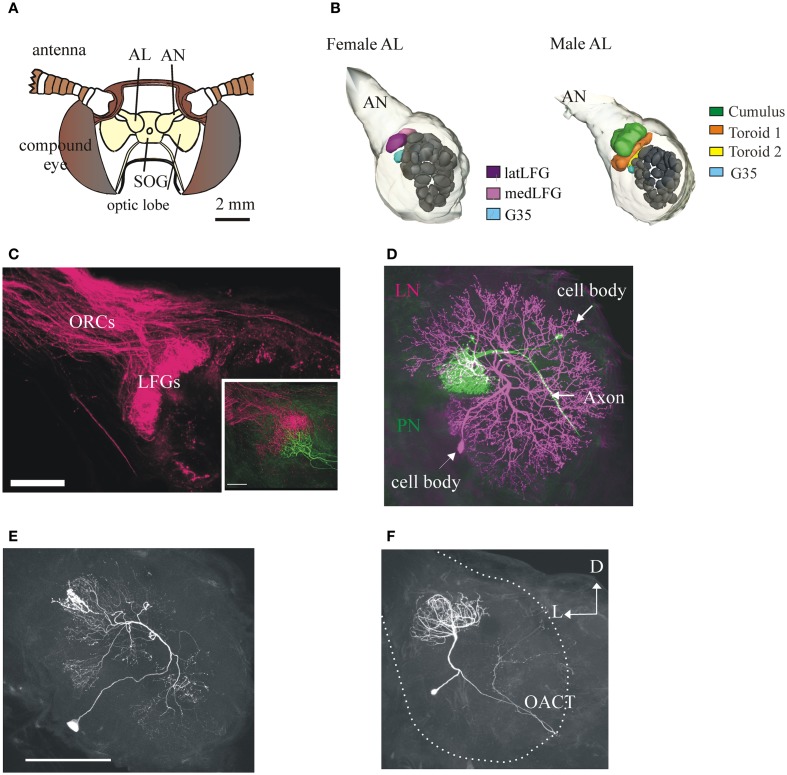
**Anatomy of the primary olfactory center of the moth ***M. sexta*** and its neuronal components**. **(A)** Schematic frontal view of the moth's head. Each antennal lobe (AL) receives input from antennal olfactory sensory neurons (OSNs) through the antennal nerve (AN) (one glomerulus receives input from the labial palps, not shown). SOG, suboesophageal ganglion. **(B)** 3-D reconstructions of the *M. sexta* ALs from a female (left) and a male (right), showing the sexually dimorphic glomeruli (LFGs in females, the Cumulus and the two Toroids in males) and a sexually isomorphic glomerulus (G35, in light blue) of known odor input. Scale bar: 50 μm. **(C)** Confocal image showing OSN afferent input to the LFGs. Inset: mass-labeled OSNs with a single-labeled LFG projection neuron (in green). Scale bar: 100 μm. Figure with permission from Dr. J. Hildebrand. **(D)** A female AL showing the two main types of neurons, a uniglomerular projection neuron (PN, in magenta) and a local interneuron (LN, in green). PNs have an axon that projects from the AL to higher brain centers in the protocerebrum; LNs are intrinsic to the AL and connect many glomeruli. Scale bar: 100 μm. **(E)** LNs are heterogeneous. A LN with arborizations restricted to relatively low number of glomeruli (compare with the LN in **D**). Scale bar: 100 μm. **(F)** The ALs also contain a sizable number of multiglomerular PNs (Homberg et al., 1988), whose functions have not been systematically studied. This PN (from a female) has arborizations in 6–8 glomeruli (including the LFGs) and projects to the protocerebrum via the outer antenno-cerebral tract (OACT) to areas clearly distinct from those where most uniglomerular terminate (compare with Figure 3A; Homberg et al., 1989). Same orientation in all panels; D, dorsal, L, lateral.

The anatomy and physiology of the olfactory system is remarkably similar across insects, including moths. Here we describe that of our exemplar herbivorous insect, the moth *M. sexta*. Antennae are the main olfactory organs of moths: the flagellum of each antenna carries thousands of cuticular hair- or peg-like olfactory organules (sensilla), each of which contains one or a few olfactory sensory neurons (OSNs) (Lee and Strausfeld, 1990). The axons of OSNs in each antennae project centrally via the antennal nerve and terminate in one of the paired primary olfactory centers in the insect brain (Tolbert and Hildebrand, 1981), the ipsilateral antennal lobe (AL) (Figure 2A). As in all insects, the ALs have numerous glomeruli, the functional modules of the AL and the first synaptic sites in the olfactory pathway (Boeckh and Tolbert, 1993; Sun et al., 1997; Figure 2B). In the fruitfly *Drosophila melanogaster* and likely in all insects, most types of OSNs expresses only one type of olfactory receptor protein (OR), and the axons of OSNs expressing the same OR converge on the same glomerulus (Gao et al., 2000; Vosshall et al., 2000). Males have OSNs which respond to the individual components of the con-specific female sex pheromone (Kaissling et al., 1989), but in some moth species certain plant odorants chemically unrelated to the sex pheromone can activate the male-detecting sex pheromone pathway at both the peripheral and the central level (Rouyar et al., 2015). The antennae of *M. sexta* also have OSNs which respond to volatiles emitted by host plant foliage, including aliphatic, aromatic, and terpenoid compounds bearing a variety of functional groups (Shields and Hildebrand, 2001; Späthe et al., 2013; **Figure 7**). The labial palps of moths of both sexes also have OSNs that respond to changes in ambient CO_2_(including floral CO_2_), a cue that is important in moth behavior, and converge in a single glomerulus in each AL (Guerenstein et al., 2004; Thom et al., 2004; Goyret et al., 2008).

Initial three-dimensional reconstructions based on anatomical analysis indicated that the ALs of *M. sexta* have 63 ± 1 identifiable glomeruli (Rospars and Hildebrand, 1992, 2000) (Figure 2B), some of which were characterized physiologically (Christensen and Hildebrand, 1987; Roche King et al., 2000; Guerenstein et al., 2004; Reisenman et al., 2004, 2005). More recent studies conducted using a non-histochemical approach based on confocal laser scanning microscopy followed by computer-assisted 3D reconstruction indicate that there are actually 70 ± 1 glomeruli in females and 68 in males (Grosse-Wilde et al., 2011). As in other moths (e.g., Berg et al., 2002), the majority of glomeruli (ca. 60) are sexually isomorphic (Figure 2B) and are involved in the processing of olfactory information about plant VOCs and perhaps other odors (e.g., Figure 4C; Guerenstein et al., 2004; Lei et al., 2004; Reisenman et al., 2005; Hillier and Vickers, 2007; Riffell et al., 2009a,b; Varela et al., 2011). A second AL subsystem comprises three male-specific glomeruli (the so-called macroglomerular complex) which process information about the conspecific female's sex pheromone (Figure 2B; Christensen and Hildebrand, 1987; Heinbockel et al., 1999, 2004). Females have a pair of large female-specific glomeruli (LFGs, Figures 2B,C, 4A,B) which respond to particular host plant VOCs (Roche King et al., 2000; Reisenman et al., 2004) and are involved in mediating oviposition behavior (Kalberer et al., 2010), and three smaller female-specific glomeruli (Grosse-Wilde et al., 2011). Correspondingly, three male-specific and five female-specific OR genes have been described in *M. sexta* (Grosse-Wilde et al., 2011). Moreover, sequence data indicates that homologous female-specific OR genes exist in different moth families (Grosse-Wilde et al., 2011), indicating that certain VOCs are important for mediating oviposition behavior across distant species.

**Figure 3:**
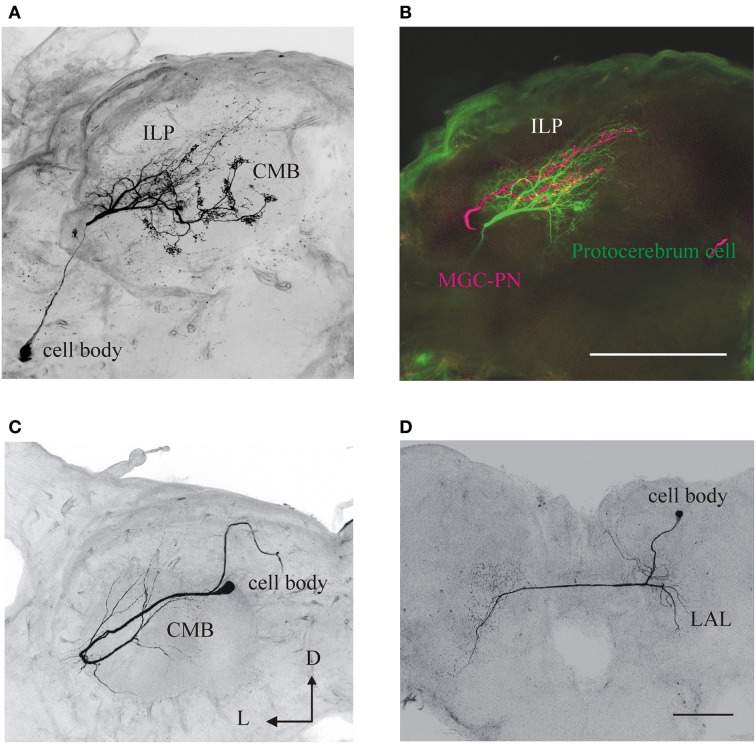
**Anatomy and morphology of olfactory neurons arborizing in second and third-order brain centers in ***M. sexta*****. These neurons might respond to other sensory modalities. **(A)** A protocerebral neuron with dendritic arborizations in the inferior lateral protocerbrum (ILP) and the calyx of the mushroom bodies (CMB). **(B)** In the same preparation a male-specific (MGC) neuron was differentially stained (in magenta) showing that both neurons have arborizations in overlapping areas (yellow). Scale bar: 100 μm. D, dorsal; L, lateral. **(C)** An olfactory-responsive neuron with arborizations in the CMB. **(D)** A neuron in the lateral accessory lobe (LAL), an area which receives input from olfactory protocerebral neurons. Scale: 200 μm. Same orientation in all panels; D, dorsal; L, lateral.

**Figure 4:**
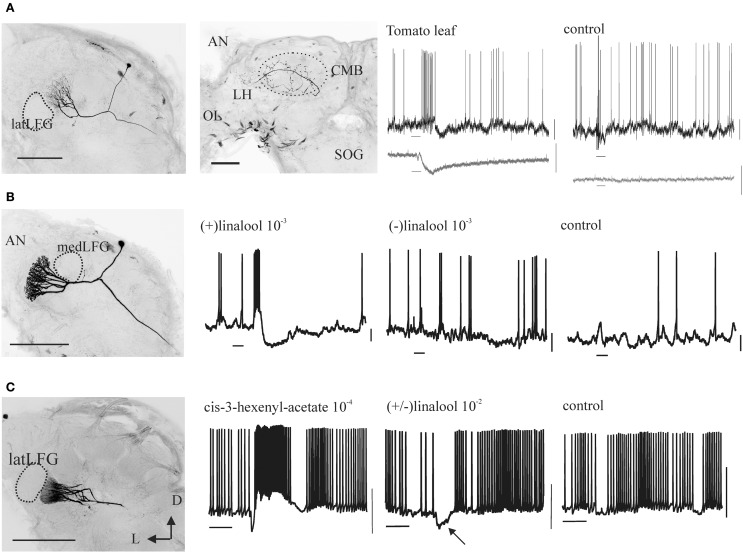
**Morphology and odor responses of female-specific antennal lobe PNs (A: medLFG-PN, B: latLFG-PN) and of a sexually isomorphic PN in the adjacent glomerulus G35 (C)**. Scale bars: 200 m. D, dorsal; L, lateral. The second panel in **(A)** shows the projection sites of LFG-PNs in the protocerebrum, the lateral horn (LH) and the calyx of the mushroom body (CMB); the optic lobe (OL) and the suboesophageal ganglion (SOG) are shown for reference. Most uniglomerular PNs also project to these sites. Shown are intracellular recordings obtained from these neurons to stimulation (duration = 200 ms, bars below records) with vegetative material **(A)** or odors **(B,C)** at the concentrations (% vol/vol) indicated. In **(A)** the bottom traces (in gray) show the simultaneously recorded electroantennograms (EAGs). Control stimuli are air from an empty cartridge **(A)** or the mineral-oil solvent **(B,C)**. Calibration bars: 10 mV (intracellular trace), 1 mV (EAG). Note that medLFG-PNs are excited by tomato leaf volatiles, latLFG-PNs respond differentially to the two linalool enantiomers, and G35-PNs are excited by *cis*-3-hexenyl acetate and hyperpolarized by (±)-linalool (arrow), i.e., by input to the adjacent latLFG.

As in many other insects, two classes of AL neurons have been identified in *M. sexta*: local interneurons (LNs; *n* ≈ 360) and projection neurons (PNs; *n* ≈ 800) (Figures 2D–F, 4). Many studies indicate that the architecture and function of AL neurons is remarkably similar among moths (e.g., Hartlieb et al., 1997; Lei and Hansson, 1999; Kanzaki et al., 2003; Seki and Kanzaki, 2008; Namiki and Kanzaki, 2011). Most PNs have dendritic arborizations restricted to a single glomerulus and an axon projecting to higher brain centers (Homberg et al., 1988). Some PNs arborize in multiple glomeruli (Figure 2F), and it is likely that they process information about particular odor blends (Heinbockel et al., 1999). The LNs receive input from OSNs, have dendritic arborizations restricted to the AL, interconnect few or many glomeruli (Figures 2D,E; Matsumoto and Hildebrand, 1981; Reisenman et al., 2011), and interact synaptically (mainly through inhibition, but see Olsen et al., 2007) with other AL neurons (Christensen et al., 1993; Reisenman et al., 2008). The major targets of PN axons are the lateral horn of the protocerebrum (PC), the inferior lateral PC, and the calyces of the ipsilateral mushroom body (Figures 3A–C, 4A; Homberg et al., 1988, 1989). Neurons in these higher-order brain centers integrate information about different odor compounds (Kanzaki et al., 1991; Lei et al., 2013; an example is shown in Figure 3C) and are involved in learning and memory (Davis, 2004; Fahrbach, 2006). Although better characterized in males, downstream neurons in the lateral accessory lobe and ventral protocerebrum (an example is shown in Figure 3D), which are thought to be main target of olfactory-responding protocebral neurons, mediate the moth characteristic olfactory-evoked sequential zigzag turns (Kanzaki and Shibuya, 1992).

Centrifugal neurons perform several important functions in the moth brain by linking different neural networks and modulating neural circuits that together lead to important physiological and behavioral responses. In particular, a small number of large aminergic centrifugal neurons have important behavioral effects. For instance, fibers from octopamine-immunoreactive neurons are found in the AL, mushroom bodies, and the lateral protocerebrum (Dacks et al., 2005); similarly, fibers from dopaminergic and serotoninergic neurons are also found in the AL and other higher brain areas, including the lateral horn. These neuromodulators increase odor-evoked responses in the majority of antennal lobe PNs and LNs, but can also decrease responses in a smaller subset (Dacks et al., 2008, 2012). Thus these neuromodulators can serve to increase the gain and sensitivity of the neural ensemble in the AL—an important feature for the moths when flower and host plants are temporally and spatially dynamic.

## Butterflies and moths: more similar than different

Among herbivorous insects, searching for a suitable host plant may involve input from different sensory modalities (Schoonhoven et al., 2005). However, the importance of olfactory cues in host finding maybe a more generalized phenomenon among the Lepidoptera than previously thought. It has long been assumed that butterflies, which are adapted to a diurnal lifestyle, use mostly visual cues to find host plants. Recent studies in the comma butterfly *Polygonia c-album*, however, showed that the anatomical and physiological characteristics of their olfactory system are remarkably similar to that of moths, despite more than 100 million years of divergence (Carlsson et al., 2011). For instance, the numerical glomeruli composition of AL of this butterfly species is comparable to that of moths, AL neurons faithfully respond to host plant extracts and plant-derived compounds, and odor-evoked AL responses match well described features such as unique and overlapping patterns of activated glomeruli (Carlsson et al., 2011). Also, studies in the butterfly *Pieris rapae*, which has an extremely well developed visual system, showed that insects can distinguish a host from a non-host plant based solely on olfactory cues (Ikeura et al., 2010).

Another interesting study compared the neural representation of plant-derived odorants in five moth species belonging to two phylogenetically distant families (Sphingidae and Noctuidae). While moths in these two families shared some (but not all) foraging and oviposition characteristics, the basic AL mapping of host plant odorants was comparable across species. Thus, these results demonstrate that similar coding strategies are used even by families separated more than 65 million years ago (Bisch-Knaden et al., 2012).

## Putting it all together: plant chemical signals, neurons, and behavior

Using *M. sexta* as an exemplary, in this section we present our current knowledge on the neural processing of relevant, naturally occurring host plant signals at several levels of the olfactory pathway, and its consequences for natural behavior. As we mentioned before, the sacred *D. wrightii* and the nocturnal moth *M. sexta* form a pollinator-plant and herbivore-plant association (Bronstein et al., 2009), with females using the plant both as a nectar (Alarcón et al., 2008; Riffell et al., 2008b) and as an oviposition resource (Mechaber and Hildebrand, 2000). Correspondingly, feeding and oviposition behaviors often co-occur in gravid females (Bronstein et al., 2009; Reisenman et al., 2010). What are the floral and vegetative VOCs that guide these behaviors, and how are they processed in the moth brain? Although *D. wrightii* flowers produce a bouquet composed of more than 60 odorants (Raguso et al., 2003) a blend of just three floral components [(±)-linalool, benzaldehyde, and benzyl alcohol], presented in appropriates ratios and concentrations, is an effective mimic of the floral scent, eliciting feeding behavior in naïve moths of both sexes (Riffell et al., 2009b). Although adult moths are innately attracted to the *D. wrightii* floral scent, they readily learn to feed on other nectar sources through olfactory conditioning (Riffell et al., 2008b).

**Figure 5:**
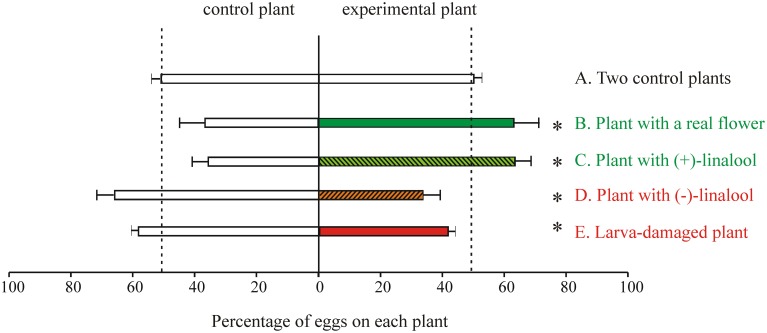
**Oviposition behavior of ***M. sexta*** in the laboratory**. In all cases single mated females were offered a choice between a control and a test plant in a flight tent (as shown in Figure 1C) and allowed to oviposit during 10 min after take-off. Plant pairs of the jimsonweed *D. wrighttii* **(A–D)** or tomato (*Solanum lycopersicum*) **(E)** were used. In**(A)** two control plants were offered to control for spatial asymmetries (*n* = 25). The following experimental series were conducted: **(B)** a plant with a newly opened flower vs. a plant with a paper flower (*n* = 12); **(C,D)** a plant with a paper flower loaded with (+)-linalool (**(C)**, *n* = 16) or (–)-linalool (**(D)**, *n* = 16) vs. a plant with a paper flower loaded with solvent (linalool was loaded in the paper flowers at the naturally-occurring concentrations); **(E)** a larva-damaged plant vs. an intact plant (*n* = 38). Data represent the percentage (average ± SE) of eggs oviposited in each plant. Moths and plant pairs were used only once. Asterisks indicate significant differences (*p* < 0.05; Sign tests). Green-hue and red-hue colors, respectively indicate oviposition attraction or repellence for the experimental plant (Data modified from Reisenman et al., 2010, 2013).

While OSNs in the female antenna of *M. sexta*, as in other moths (e.g., Hillier et al., 2006; Ulland et al., 2008), respond to a chemical variety of host plant VOCs (Figure 7; Shields and Hildebrand, 2001; Späthe et al., 2013), we found that (±)-linalool, a floral volatile characteristic of many moth-pollinated night-blooming flowers including *D. wrightii*, has important roles mediating behavior (Riffell et al., 2008b, 2009a; Reisenman et al., 2010, 2013). Behavioral and electrophysiological recordings from AL-PNs showed that the two naturally occurring enantiomers of linalool present in flowers mediate feeding and oviposition through two neural pathways, one that is sexually isomorphic and non-enantioselective, and another that is female-specific and enantioselective (Figures 4B, 5; Reisenman et al., 2004, 2010). In one hand, the (+) and (−) enantiomers of linalool respectively contribute to oviposition attraction and repellence and are discriminated by female-specific PNs (Figures 1C, 4, 5). Linalool-responsive sexually isomorphic PNs do not discriminate between linalool enantiomers (Reisenman et al., 2004) and correspondingly, the enantiomers are not discriminated in the feeding context (Reisenman et al., 2010). Interestingly, two homologous receptors to the *Bombyx mori* linalool-ORs, MsexOR-5 and 6, have been described in *M. sexta* (Grosse-Wilde et al., 2010, 2011), and are likely candidates to mediate (at least in part) these behaviors. This, together with the fact that these moth species belong to evolutionary distant families, suggest that these receptors and the corresponding neurons play an important role in moth, and probably Lepidoptera, olfaction (Grosse-Wilde et al., 2011).

**Figure 6:**
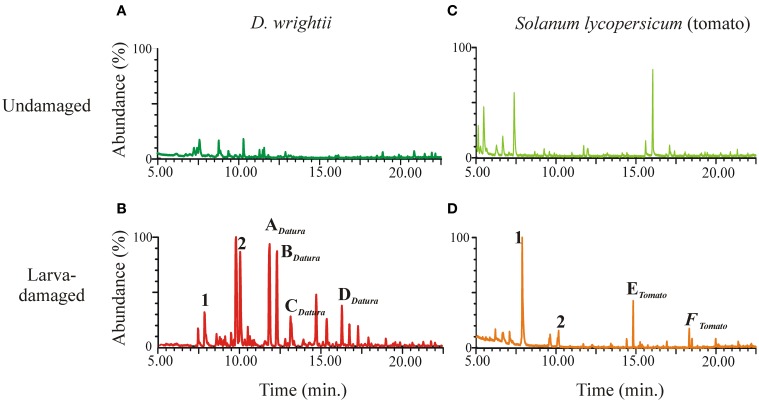
**Plant VOC response to herbivory**. ***M. sexta*** larvae were allowed to feed on plants during 2–3 days, after which larvae and frass were removed and vegetative VOCs collected and analyzed via GC-MS as described in Reisenman et al. (2013). Representative ion chromatograms of the headspace from undamaged *D. wrightii* **(A)**, larva-damaged *D. wrightii* **(B)**, undamaged tomato **(C)**, and larva-damaged tomato **(D)**. In **(C,D)** same numbers or letters, respectively indicate common and different compounds emitted by larva-damaged vegetation.

**Figure 7:**
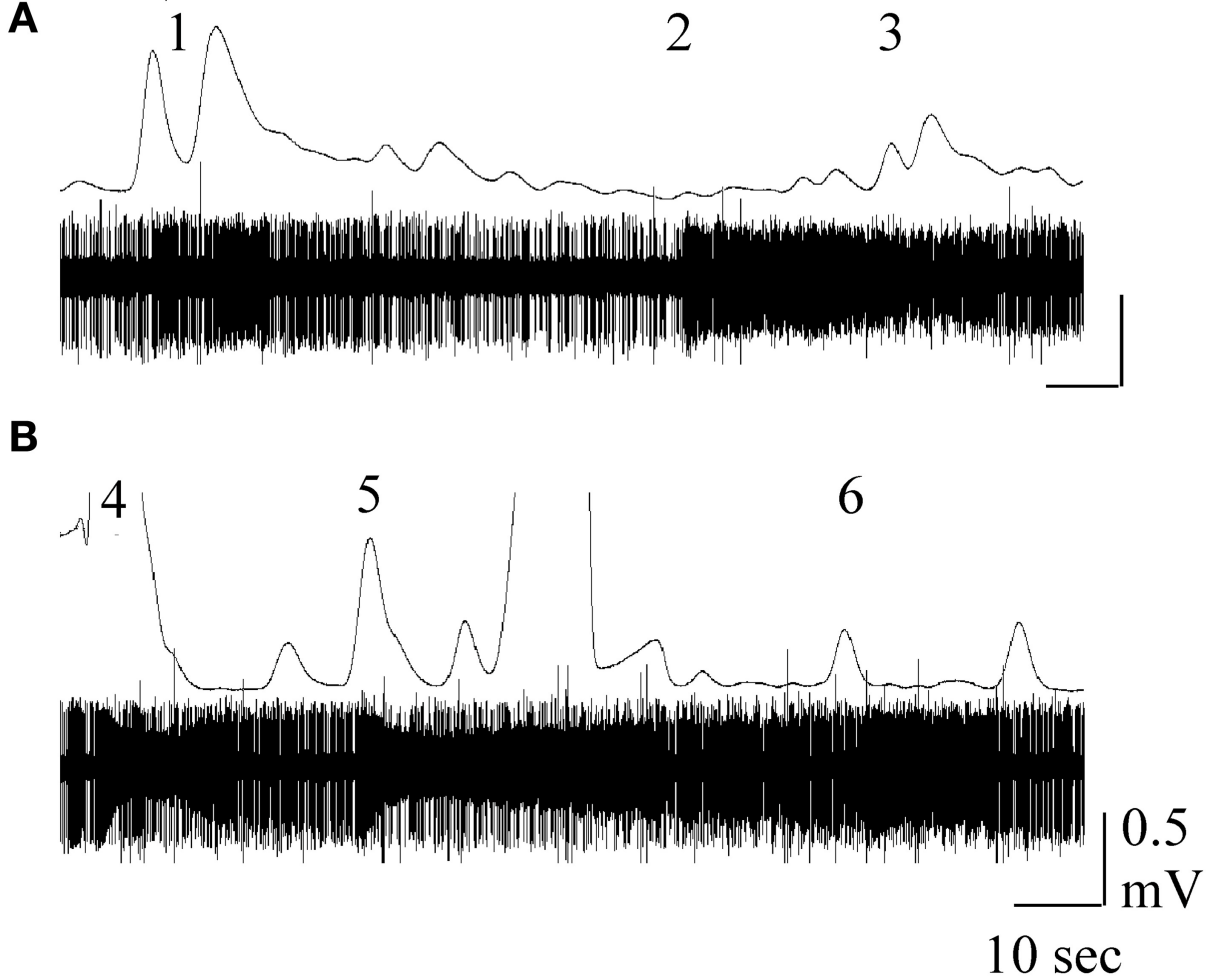
**Simultaneous GC trace (top) and single sensillum recording (bottom trace) obtained in response to stimulation with ***M. sexta*** larva-damaged tobacco (*Nicotiana attenuata*, A) or *D. wrightti* (B)**. Numbers indicate neuron responses to phenol (1), 4-methyl pentanol (2), *cis*-3-hexanol (3), cis-3-hexenyl acetate (4), benzyl alcohol (5) and methyl-benzoate (6). For methods, see Späthe et al. (2013). Figure courtesy of Drs. S. Kesevan and S. Olsson.

While *M. sexta* uses a variety of host plants for oviposition, choice experiments showed that females prefer to oviposit on *D. wrightti* plants, and that this preference is mostly mediated by olfactory cues (Späthe et al., 2013). Although females avoid ovipositing in larva-damaged plants (Figure 5E), this avoidance is plant- specific: females strongly avoid larva-damaged tomato and tobacco plants, but they do not avoid ovipositing in larva-damaged *D. wrightti* plants, despite that these plants can be clearly distinguished from non-damaged plants by their VOC profile and by the peripheral OSNs (Figures 6, 7; Reisenman et al., 2013; Späthe et al., 2013). An important consideration is that moths use these plant species differently: while the annuals tomato and tobacco are only used by moths for oviposition, the jimsonweeds are also pollinated by the adults. Thus, we propose that the differences in oviposition preference toward larvae-damaged plants of different species are due to the different relationships between *M. sexta* and these host plants. The beneficial association is emphasized further by the finding that at least some of the *D. wrightii* floral VOCs that are important to mediate feeding– and hence pollination–remained unchanged in herbivore-induced plants (Reisenman et al., 2013).

As we mentioned before, (+)-linalool has an important role in mediating oviposition attraction. In contrast, we found that plants with (−)-linalool added are avoided by ovipositing females (Figure 5D; Reisenman et al., 2010), and that this compound is significantly increased in larva-damaged tomato plants (Reisenman et al., 2013). The antenna of the cabbage moth *Mamestra brassicae* is, as that of *M. sexta* (Reisenman, not shown), more sensitive to (−)-linalool than to (+)-linalool (Ulland et al., 2006). Collectively, these results suggest that (−)-linalool (alone or together with other with other induced VOCs; Reisenman et al., 2013), might act as an oviposition repellent and also as a plant defense, attracting the natural enemies of herbivores (Baldwin et al., 2002). The finding that this unique odorant is similarly processed and discriminated by moths in different families also suggests that common components and neural mechanisms are involved in the selection of suitable host plants.

**Figure 8:**
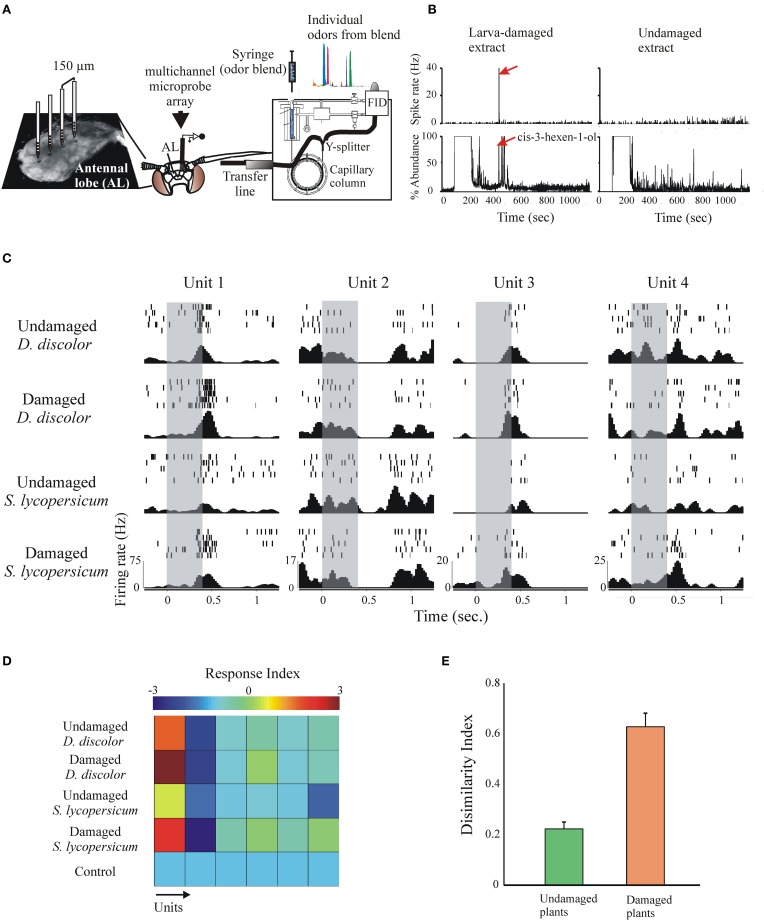
**The response of many simultaneously recorded AL neurons to VOC extracts and single components can be studied using a multi-unit recording electrode coupled to gas-chromatographic analysis (GC-MRA)**. **(A)** Plant extracts are injected on the GC inlet; after leaving the GC column a Y-splitter divides the effluent for simultaneous antenna stimulation and chemical identification of individual components. **(B)** GC-MRA analysis showing a neuron with robust responses to the larva-damaged VOCs responding selectively to only one peak in the GC effluent (arrow, cis-3-hexen-1-ol). **(C)** Simultaneously recorded responses of 4 units to stimulation (duration = 200 ms, gray bars) with the plant extracts indicated to the left. Shown are individual spikes (tick marks) during repetitive stimulation (rows), and the peri-event histograms calculated across trials (bottom). Note that different units respond differently to different stimuli, and that the same unit responds differentially to intact and larva-damaged extracts. **(D)** Response indexes (or z-scores, color-coded, calculated as spiking rate during stimulation—spiking rate pre-stimulation/SD) for six simultaneously recorded neurons in response to stimulation with the extracts indicated. The first two units showed stronger responses to stimulation with larva-damaged plants. **(E)** The dissimilarity index (Riffell et al., 2009a) indicates stronger AL neuronal ensemble responses to larva-damaged plants.

Although linalool has important roles in mediating oviposition, it is very likely that the choice of suitable host plant sites is mediated by a suite of VOCs. A powerful technique to address this issue, which has been already used to investigate the VOCs involved in mediating feeding (Riffell et al., 2009a,b), is to couple the use of gas chromatography for chemical detection and multi-unit recordings from AL neurons (GC-MR; Figure 8A). This technique allows to simultaneously visualize the activity of many neurons in response to components from behaviorally active plant extracts as they elute from the GC column. For instance, Figure 8B shows an example of a neuron that responds specifically to a single larva-damaged component. The use of the multi-unit recording technique allows stimulating many neurons with different bioactive plant extracts (Figure 8C). Quantification of individual neuron responses to repetitive stimulation evinced plant- and status- (intact or damaged) specific responses, either excitatory or inhibitory (Figures 8C,D). To evaluate the population response, we calculated a dissimilarity index, which indeed demonstrate that the AL discriminates between larva-damaged and intact plants (Figure 8E). Knowledge of the compounds that are discriminated at this level of olfactory processing readily informs about the suite of VOCs that could potentially mediate the behavioral selection of appropriate host plants.

## Olfactory responses in sexually isomorphic pathways and interconnected glomeruli

As described above, female specific neurons are involved in mediating female-specific behaviors such as oviposition (Roche King et al., 2000; Reisenman et al., 2004) and in some moth species, detection of male pheromones (Hillier et al., 2006). As in many insects, the orientation of males toward the female-specific sex pheromone is crucial for the species survival, and the role of male-specific neurons in mediating this behavior is well-established in many moth species (e.g., Anton et al., 1997; Berg et al., 1998; Lei and Hansson, 1999; Vickers and Christensen, 2003). Although it might be tempting to argue that sexually dimorphic pathways are particularly selective as they mediate fundamental behaviors related to reproduction, we found that PNs in sexually isomorphic glomeruli can also be highly specific. For instance, PNs in an identified glomerulus (glomerulus 35, which neighbors the sexually dimorphic glomeruli in both sexes, Figure 2B) are extremely selective and sensitive to another host plant volatile, cis-3-hexenyl acetate (Figure 4C), responding to concentrations < 1 ppm (Reisenman et al., 2005). While the specific role of this VOC for behavior is not yet elucidated (although its production is augmented in larva-damaged plants; Hare, 2007), knowledge of the specific VOCs that activate specific sets of glomeruli has provided a tool to study interactions between glomeruli involved in mediating different behaviors. Previously, Lei and coworkers elegantly demonstrated that the temporal output of each male-specific glomerulus is enhanced by reciprocal inhibitory interglomerular interactions, and that this serves to synchronize the activity of neurons processing the components of the sex pheromone blend (Lei et al., 2002). Using known odor inputs to activate specific glomeruli beyond the sex-specific system, we found that the two AL subsystems interact synaptically in a distant-independent, non-reciprocal fashion (Figure 4C, middle panel; Reisenman et al., 2008), and that these interactions are mediated by a functionally and morphologically heterogeneous population of local interneurons (Figures 2D,E; Reisenman et al., 2011). Interactions between odors with different behavioral significance have been also described in other moth species, both at a behavioral and at a neural level (e.g., Chaffiol et al., 2012, 2014; Deisig et al., 2012; Trona et al., 2013).

Experiments conducted in many insect species, including *M. sexta*, indicate not only that glomeruli interact synaptically, but that sets of interconnected glomeruli are likely involved in the processing of behaviorally relevant odor blends. At the AL level, this idea is supported by the fact that a sizeable proportion of LNs interconnect a restricted subset of glomeruli (Figure 2E; Reisenman et al., 2011). The existence of PNs that arborize in multiple -but restricted- glomeruli, also supports this hypothesis (Figure 2F). At the level of the chemical signals, it is known that some of the active compounds identified in the host plant headspace are ubiquitous floral and vegetative VOCs. Thus, it is possible that a suite of compounds presented in particular proportions (Thiery and Visser, 1986; Zhang et al., 1999; Riffell et al., 2008b, 2009a), rather than a single compound, activates a subset of glomeruli to mediate host plant selection. For instance, a blend of just three floral *D. wrightii* VOCs (but not any of the single VOCs) can elicit feeding (Riffell et al., 2009b). Similarly, the sole presence of linalool is not sufficient to mediate oviposition, although the presence of this component in plants has profound behavioral effects (Figure 5; Reisenman et al., 2010). Because different host plants are readily accepted for oviposition by females, it is possible that that individual VOCs shared across plant species activate a functionally connected glomerular subset (which necessarily involves at least some of the female-specific glomeruli), the output of which ultimately control oviposition behavior. The chemical composition of that bouquet, however, remains to be identified.

## Moths find plants, but how do the plants impact the moths?

While in the previous section we discussed the neural processing of naturally occurring signals and its consequences for behavior, in this section we highlight some plant cues and signals that in turn, can influence moth behavior. From the plant prospective, what matters is to attract efficient pollinators. In the case of *D. wrightti*, as we mentioned, this has the un-intended consequence of also attracting gravid females. This plant species can effectively cope with this, as plants can tolerate high levels of defoliation, quickly regrow after herbivory, reduce photosynthetic rates, and redirect resources to storage in the roots upon herbivory (cited in Reisenman et al., 2010). However, once moths probe flowers, other gustatory sensory cues present in nectar appear to have profound effects in guiding behavioral decisions.

As mentioned before, plants often produce secondary compounds (e.g., alkaloids, glycosides, and phenolic compounds) to deter herbivores and pathogens (Karban and Baldwin, 1997). Interestingly, these secondary compounds are also present in floral nectar (Adler, 2000) and can be induced by herbivory in a plant-species specific manner (Adler et al., 2006; Hare and Walling, 2006; Kessler and Halitschke, 2009). It has been suggested that the presence of these components in nectar increases pollinator fidelity, repel nectar robbers, and improve pollen transfer by intoxicating pollinators (Adler, 2000).

In the case of *D. wrightii*, an obvious advantage for female *M. sexta* is that visits to flowering plants provide both nectar and oviposition resources. However, because of the limited availability of the flowers (which bloom for only one evening) and the effects of herbivory (which limits the number of healthy host plants), female moths encounter resources that are spatio-temporally patchy. The moth nervous system has the ability to adjust its activity so that behavioral output is maximally beneficial for survival within an often harsh and patchy environment. This plasticity is accomplished by the release of specific neuromodulators—including serotonin, octopamine and dopamine—within restricted brain regions like the AL, the lateral horn and the mushroom bodies. For example, herbivory-induced damage to *D. wrightii* plants elicits high concentrations of certain alkaloids in the flower nectar which *M. sexta* finds aversive. Tropane alkaloids (including scopolamine) in the nectar of damaged *D. wrightii* increase more than 20-fold in damaged plants (Dacks et al., 2012). Over time, moths learn the association between the alkaloid content and the floral and vegetative scent so that these damaged plants become avoided. Dopamine release in the moth brain—including the ALs and the mushroom bodies—has shown to be a critical signal mediating aversive learning and signaling the presence of an aversive stimulus. For instance, when the ALs of female moths were injected with a dopamine receptor antagonist, moths could no longer learn the association of the aversive nectar and the flower scent. Furthermore, when dopamine was superfused on to the AL, the neural ensemble showed enhanced responses to the flower odor stimulus. Dopaminergic modulation of AL circuits thus plays an important role in the memory formation of repellent flower scents and the discrimination of larva-damaged plants. Interestingly, the effects of alkaloids in moth preference are shaped by both the plant species and the behavioral context. For instance, females prefer to oviposit in tobacco plants which have higher concentrations of nicotine in nectar (Adler et al., 2006), but they remove less nectar from these plants (Kessler and Baldwin, 2006). Thus, the nervous system can differentially evaluate the same plant sensory cue (in this case, nicotine) according to the behavioral context.

What are the effects of nectar secondary compounds on insect behavior? In general, naturally-occurring concentrations of secondary compounds do not deter nectar-feeding insects, whether specialists or generalists. In generalist insects such as bees, low concentrations of certain secondary compounds such as nicotine and caffeine elicit feeding preference (Singaravelan et al., 2005). This preference is not mediated by peripheral taste receptors, but is probably due to the effect of these substances in reward brain centers (Singaravelan et al., 2005; Kessler et al., 2015). In contrast, prolonged exposure to high concentrations of these compounds (e.g., such as those found in flowering crops sprayed with neonicotinoid pesticides) can impair olfactory learning and memory (Williamson and Wright, 2013). The effects of these substances in learning and memory in herbivorous insects which are exposed to natural concentrations of plant secondary defenses have not been yet studied, but tobacco plants which have been engineered to completely lack nicotine in nectar have more nectar removed per night (Kessler and Baldwin, 2006). Unlike bees, taste receptors in the mouthparts of moths can readily detect bitter compounds such as caffeine (Bernays et al., 2002; Glendinning et al., 2006), which are commonly present in their host plants. The effects of these substances on behavior, however, remain to be investigated in the appropriate ecological and behavioral context.

## Summary and conclusions

In the last couple of decades, research in the neuroscience field has focused on a small number of “model” species offering various advantages, at the expense of potentially creating a bottleneck which limits or compromises our understanding of how nervous systems operate (Brenowitz and Zakon, 2015). Today, several genome project efforts, and increasingly available tools that allow DNA editing, are bridging this gap. However, an integrative approach that includes ecological and community relationships, natural signals, neurons and behavior, has always been and it will always remain key to understand the function of nervous systems.

Here we used an exemplary specialized herbivorous insect, the moth *M. sexta*, to review the function of the moth's olfactory system in a naturalistic context. While floral odors attract moths for feeding and oviposition, volatiles released from larva-damaged plants mediate oviposition repellence. Specific plant volatiles are involved in mediating these behaviors, and are processed in both sexually isomorphic and dimorphic neural pathways according to the behavioral context (feeding and oviposition). Furthermore, some of these volatiles also mediate behavior in distant moth species, suggesting important roles for certain plant volatiles and commonalities in their neural processing. In addition, for the plant benefit, plant secondary compounds can affect host-plant finding and behavior through processes such as learning and memory.

While today we have a deeper understanding of how the nervous system process information about behaviorally relevant VOCs in *M. sexta* and other insect species, many issues, at several levels of interactions, need to be addressed. For instance, do different populations of host plants differ in their VOC profile, and what are the behavioral consequences? Given that *M. sexta* is found throughout a wide range in the American continent, is there a common set of VOCs that guide oviposition choice, despite that moths across the distribution range use different host plant species? Is there a minimum VOC blend that produces acceptance and egg lying? Would this blend suffice to guide oviposition choice in different moth populations? Are populations in other regions as specialized in certain host plants as the Southwest USA population? Do background odors affect olfactory-guided oviposition choices? Do other other factors such as temperature and humidity affect host plant choice? In nature, do females actually avoid ovipositing on plants where other larvae are already present, and if so, how do they achieve this? An interesting possibility, given that *M. sexta* host plants naturally produce alkaloids which can be readily detected by moths (Bernays et al., 2002; Glendinning et al., 2006), is that plants manipulate nicotine concentrations in nectar to their benefit, as these substances could act as postingestive stimulants and even have addictive properties, improving flower finding and efficiency (Singaravelan et al., 2005; Kessler et al., 2015). We know much about the “Neuroecology” of oviposition and feeding behavior in adult insects, but do larvae make choices within a plant, and how are these choices guided? Do larvae prefer younger or older leaves, small or big? Importantly, a comparative strategy has the power of help unraveling general neural mechanisms and strategies that guide host plant choice. We propose that all these issues by necessity need to be investigated in an appropriate Neuroecology framework.

Finally, along with a deep understanding of the relationships of organisms with their natural environment, we believe that in the near future genomic tools now available to many insect species will permit a deeper and more complete understanding of how the insect nervous system produces adaptive behavior.

### Conflict of interest statement

The authors declare that the research was conducted in the absence of any commercial or financial relationships that could be construed as a potential conflict of interest.
